# Widespread transposon co-option in the *Caenorhabditis* germline regulatory network

**DOI:** 10.1126/sciadv.abo4082

**Published:** 2022-12-16

**Authors:** Francesco Nicola Carelli, Chiara Cerrato, Yan Dong, Alex Appert, Abby Dernburg, Julie Ahringer

**Affiliations:** ^1^ Wellcome Trust/Cancer Research UK Gurdon Institute, Cambridge, UK.; ^2^ Department of Genetics, University of Cambridge, Cambridge, UK.; ^3^ Department of Molecular and Cell Biology, University of California, Berkeley, Berkeley, CA 94720-3200, USA.; ^4^ Howard Hughes Medical Institute, 4000 Jones Bridge Road, Chevy Chase, MD 20815, USA.; ^5^ Biological Sciences and Engineering Division, Lawrence Berkeley National Laboratory, Berkeley, CA 94720, USA.; ^6^ California Institute for Quantitative Biosciences, Berkeley, CA 94720, USA.

## Abstract

The movement of selfish DNA elements can lead to widespread genomic alterations with potential to create novel functions. We show that transposon expansions in *Caenorhabditis* nematodes led to extensive rewiring of germline transcriptional regulation. We find that about one-third of *Caenorhabditis elegans* germline-specific promoters have been co-opted from two related miniature inverted repeat transposable elements (TEs), CERP2 and CELE2. These promoters are regulated by HIM-17, a THAP domain–containing transcription factor related to a transposase. Expansion of CERP2 occurred before radiation of the *Caenorhabditis* genus, as did fixation of mutations in HIM-17 through positive selection, whereas CELE2 expanded only in *C. elegans*. Through comparative analyses in *Caenorhabditis briggsae*, we find not only evolutionary conservation of most CERP2 co-opted promoters but also a substantial fraction that are species-specific. Our work reveals the emergence and evolutionary conservation of a novel transcriptional network driven by TE co-option with a major impact on regulatory evolution.

## Teaser

One third of germline-specific promoters in Caenorhabditis derive from the co-option of two transposon families.

## INTRODUCTION

Cis-regulatory elements play fundamental roles in gene expression yet can undergo remarkably rapid evolutionary turnover (*1*–*3*). Since seminal work from McClintock (*4*) and later from Britten and Davidson (*5*), transposable elements (TEs) have been considered a potential source of novel regulatory elements. TEs often harbor regulatory sequences recognized by the host machinery, and if moved to an appropriate location, then they may affect the expression of host genes. Clear evidence for co-option of some TE insertions into host regulatory networks has been documented [reviewed in (*6*, *7*)], and it has been suggested that the amplification of a TE family could lead to the concerted co-option of many TEs, markedly changing whole regulatory networks. In support of this scenario, specific repeat families are enriched near putative cis-regulatory regions of coregulated genes in different species, and multiple lines of evidence suggest that TEs transcriptionally control the immune system, stress responses, and the physiology of complex tissues such as the germ line and the mammalian placenta (*8*–*12*). Nonetheless, the role of proposed large-scale co-option events is still uncertain [e.g., (*13*)], and there is limited functional evidence in vivo to support widespread or concerted transcriptional rewiring (*6*). Here, we show through genomic and functional analyses in *Caenorhabditis* that two independent TE expansions gave rise to promoters that control the expression of a large fraction of germline-specific genes.

## RESULTS

**Fig. 1.**
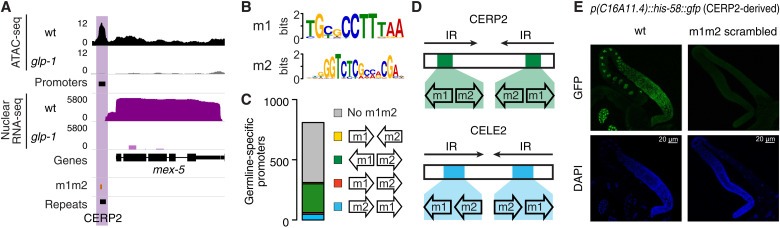
TE enrichment at germline-specific elements in *C. elegans*. (**A**) Example of germline-specific (purple) promoter in *C. elegans*. (**B**) Sequence logos of the m1 and m2 motifs. (**C**) Number of m1m2 pairs overlapping germline-specific promoters, color-coded based on their relative orientation. (**D**) Location of m1m2 pairs in CERP2 and CELE2 consensus. IR, inverted repeat. (**E**) Green fluorescent protein (GFP) and 4′,6-diamidino-2-phenylindole (DAPI) signals from CERP2-derived wild-type (wt) *p(C16A11.4)::his-58::gfp* and m1m2-scrambled *p(C16A11.4)::his-58::gfp* in adult gonads. Scale bars, 20 μm.

To investigate transcription regulation in the *Caenorhabditis elegans* germ line, we first identified germline-specific accessible chromatin sites (*n* = 2316) based on the presence of a strong Assay for Transposase Accessible Chromatin with high-throughput sequencing (ATAC-seq) signal in wild-type young adults but not in *glp-1* mutants lacking a germ line (fig. S1A) (*14*). Using nuclear RNA sequencing (RNA-seq) patterns to link open chromatin regions to annotated genes, we then classified 782 sites as germline-specific promoters (Fig. 1A and table S1; see Materials and Methods). Sequence analysis of these promoters revealed the enrichment of two motifs (m1 and m2; Fig. 1B) that do not share significant similarity with other eukaryotic regulatory motifs but were previously identified upstream of *C. elegans* genes with germline expression (*15*). We found that an m1m2 pair is present in 36.3% (284 of 782) of all germline-specific promoters; of these, 76.8% are found in a divergent orientation and 15.1% in an m2+m1+ tandem orientation (Fig. 1C). Genes associated with m1m2-containing promoters are more highly expressed than other germline genes, and their promoters show greater accessibility in primordial germ cells (PGCs) and in late larvae, which contain many germline cells (fig. S1, B and C). Promoters containing m1m2 motif pairs were also found upstream of 177 genes expressed in both germ line and soma, which predominantly show ubiquitous accessibility by ATAC-seq (fig. S1D).

While m1m2 pairs were strongly associated with germline promoters, many additional copies of these motifs were also found in nonaccessible regions of the *C. elegans* genome in both divergent (*n* = 1458) and tandem (m2+m1+, *n* = 2566) orientations. These sequences predominantly corresponded to the positions of CERP2 (*16*) and CELE2 (*17*) elements, respectively. CERP2 and CELE2 represent two families of miniature inverted repeat TEs (MITEs), small, nonautonomous elements derived from autonomous DNA transposons (*18*, *19*). The inverted repeats of both elements contain m1 and m2 motifs, divergently oriented in CERP2 and m2+m1+ tandemly in CELE2 (Fig. 1D and fig. S1E). The finding of m1m2 pairs in germline promoters and repeat elements suggests that the m1m2-containing promoters may have been derived from CERP2 or CELE2 repeats. In line with this hypothesis, many m1m2-containing germline-specific promoters overlapped a repeat annotated as CERP2 (*n* = 68) and/or CELE2 (*n* = 61), and annotated CERP2 and CELE2 elements are enriched at germline-specific promoters at genome-wide scale (permutation test, *P* < 0.001; see Materials and Methods). We note that a previous study did not find m1m2 pairs in repetitive elements because repeat sequences were masked in the analyses (*15*).

To investigate the evolutionary relationship between the m1m2 promoters and the repeat elements, we generated a tree based on multiple sequence alignments of all m1m2-containing sequences and classified them into four categories: promoter, repeat element, both promoter and repeat element, and neither promoter nor repeat element (fig. S2). The motif-containing promoters were found among nonpromoter MITE elements in both trees, supporting the view that the motif-containing promoters were derived from the MITE elements (fig. S2). On the basis of these findings and functional analysis described below, we will hereafter refer to m1m2-containing promoters as co-opted elements. Notably, both co-opted promoters and nonpromoter CERP2 elements, and to some extent the CELE2 family, contain a region of 10–base pair (bp) periodic TT bias. This feature was recently shown to be associated with strong nucleosome positioning in *C. elegans* germline promoters (fig. S1F) (*20*) and may have contributed to the co-option of these MITEs by creating a chromatin environment that facilitates transcription in this tissue. CERP2 and CELE2 MITEs thus provided the raw material for the emergence of hundreds of *C. elegans* germline-active promoters, including around one-third of all germline-specific promoters.

To functionally test the activity of MITE-derived promoters and to understand the relevance of the m1 and m2 motifs for germline transcription, we generated transgenes containing wild-type or mutant motifs. CELE2- and CERP2-derived promoters containing wild-type m1m2 sequences drove germline-specific expression of a histone–green fluorescent protein (GFP) reporter (Fig. 1E and fig. S1G). We found that both motifs were required for promoter activity, as GFP was not detectable after scrambling m1 or m2 (Fig. 1E and fig. S1G). We identified an additional motif (m3, table S1)—found in most germline-specific promoters, often alongside the m1m2 pair, but found that it was not required for germline expression. To test motif requirements at an endogenous locus, we used CRISPR-Cas9 editing to scramble m1 and m2 in the CERP2-associated *T05F1.2* promoter and found that this reduced expression by 5.9-fold (fig. S1H). These results show that co-opted promoters drive germline expression and that the motif pairs are needed for promoter activity. Notably, as for *T05F1.2*, around 60% (171 of 284) of germline-specific co-opted promoters are the only promoters for the associated genes. Thus, the co-opted promoters are required for the expression of hundreds of germline-specific genes.

To identify potential transcription factors that might regulate co-opted promoters, we analyzed modERN and modENCODE transcription factor binding data (*21*) for enrichment at co-opted versus non–co-opted germline promoters. We found that HIM-17 showed the highest enrichment (>7.6-fold; fig. S3A). HIM-17 is a germline chromatin–associated factor important for diverse germline functions including the proliferation versus meiotic entry decision, meiotic double-strand break (DSB) formation, and germline chromatin modification (*22*–*25*). It has six THAP domains, putative sequence-specific DNA binding domains shared by P-element family transposases (*26*).

**Fig. 2.**
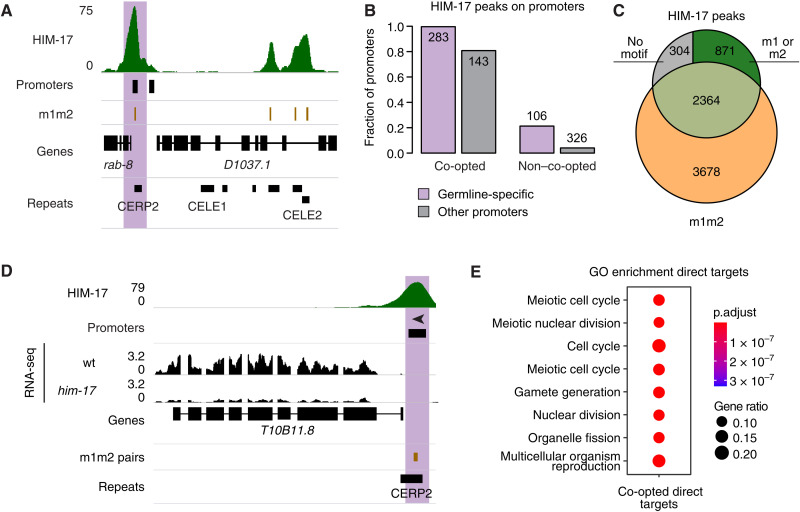
HIM-17 binds and regulates co-opted MITEs. (**A**) Example of HIM-17 ChIP-seq binding profile. (**B**) Fraction of promoters overlapped by HIM-17 peaks. (**C**) Genome-wide overlap between HIM-17 peaks and m1m2 pairs. (**D**) Example of a gene down-regulated specifically in *him-17* mutants. (**E**) Gene Ontology (GO) term enrichment of HIM-17 down-regulated direct targets regulated by a co-opted promoter.

As the HIM-17 chromatin immunoprecipitation sequencing (ChIP-seq) modENCODE data were from a mutant background, we generated new HIM-17 ChIP-seq data from wild-type adults, which identified 3539 HIM-17 peaks (Fig. 2A and table S1). HIM-17 binding was strongly associated with m1m2 motifs (table S1); all but one of the 284 co-opted germline-specific promoters was associated with a HIM-17 peak, as were 80.8% of nongermline–specific co-opted promoters (Fig. 2B). Overall, 66.8% of the HIM-17 binding sites were associated with an m1m2 pairs. Most of these were located in a closed chromatin environment overlapping an CELE2 or CERP2 element (Fig. 2C and fig. S3B). An additional 24.6% of HIM-17 peaks significantly overlapped at least an individual m1 or m2 motif (permutation test, *P* < 0.001; Fig. 2C) and had weaker ChIP-seq signals (fig. S3C). The m1m2 pair is the likely determinant of HIM-17 binding, as HIM-17 enrichment at a co-opted promoter was abolished when either m1 or m2 was mutated (fig. S3D).

To determine whether HIM-17 plays a role in co-opted promoter activity, we analyzed gene expression in young adults of the strong loss-of-function mutant *him-17(me24)*, which identified 1311 up-regulated and 1640 down-regulated genes (fig. S3E and table S1). We then defined 304 direct targets of HIM-17 as misregulated genes with HIM-17 binding in wild-type adults. Direct targets were largely down-regulated in the mutant, suggesting that HIM-17 is a transcriptional activator (Fisher’s exact test, *P* < 10^−15^; Fig. 2D, fig. S3E, and table S1). Most direct targets (193 of 304, 63.4%; table S1) are regulated by a co-opted promoter, showing that HIM-17 directly controls the transcription of a large fraction of germline genes whose promoters were co-opted from MITEs. Many of the remaining promoters are “HOT” (highly occupied target) regions, which are thought to represent nonsequence-specific transcription factor binding or ChIP artifacts, and so are unlikely to be directly regulated by HIM-17 (fig. S3F) (*21*, *27*). The significant but modest reduction in expression of most direct targets observed in the *him-17(me24)* mutant (table S1) suggests that additional transcription factors may also contribute to the expression of these genes.

Direct targets regulated by co-opted promoters are enriched for genes involved in meiosis and reproduction (Fig. 2E and fig. S3G), consistent with *him-17* mutant phenotypes (*22*). Among the genes strongly down-regulated in *him-17(me24)* mutants are *him-5* and *rec-1* (fig. S3H), two paralogs that promote DSB formation during meiosis (*28*). Down-regulation of these HIM-17 targets likely accounts for the strong reduction in DSBs and the resulting high incidence of males (Him) phenotype observed in *him-17* mutants (fig. S3E) (*22*, *25*). The pleiotropic effects on germline processes observed in *him-17* mutants (*22*–*25*, *29*), a factor that directly controls the activity of many co-opted promoters, highlight the biological impact of the co-option of hundreds of germline promoters in *C. elegans*.

**Fig. 3.**
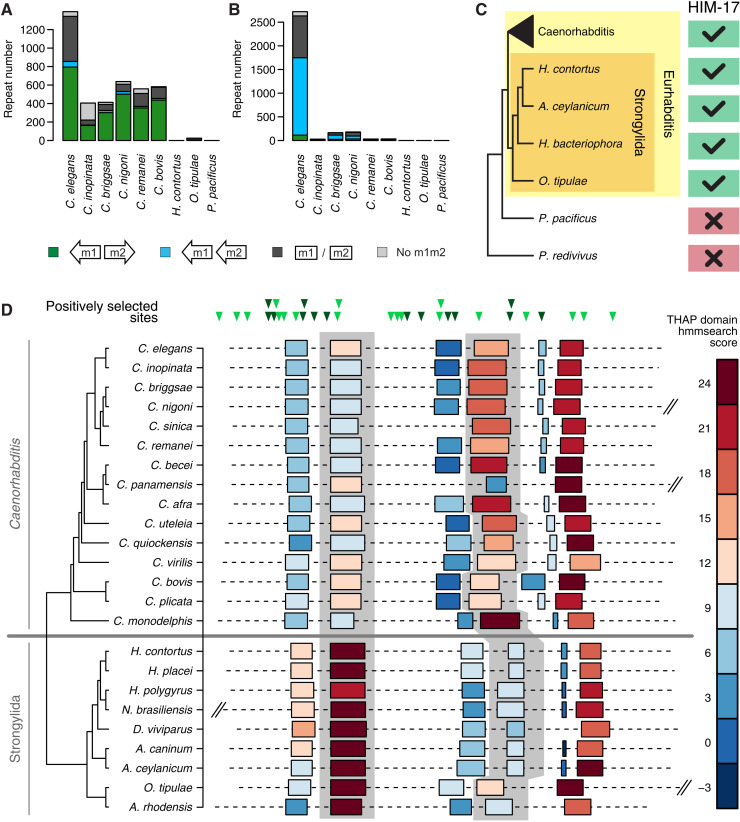
Evolution of m1m2 pairs and their binding factor in nematodes. (**A** and **B**) Number of CERP2 (A) and CELE2 (B) elements annotated in the genomes of different nematode species, and fraction of divergent m1m2 pairs, tandem m2 m1+ pairs, and other m1 and or m2 motifs overlapped. (**C**) Evolutionary conservation of *him-17*. (**D**) Top: Location of sites under positive selection with respect to the *C. elegans* HIM-17 protein. In dark, sites located in a THAP domain. Bottom: Location of THAP domains in HIM-17 orthologs. Color code reflects their similarity to the canonical THAP domain (based on the hmmsearch score). Second and fourth THAP domains are highlighted in gray. Protein length is drawn to scale and truncated for longer orthologs.

To gain further insights into CERP2 and CELE2 co-option and their regulation by HIM-17, we investigated their evolution through comparative analyses in nematodes. We first sought to determine the timing of the co-option by dating the TE expansion events. CERP2 elements are abundant in the genomes of all *Caenorhabditis* species that we analyzed but not in other nematodes (Fig. 3A). In contrast, CELE2 elements were detected only in *C. elegans*, suggesting a recent, species-specific expansion of this repeat family (Fig. 3B). The earlier expansion of CERP2 is also reflected in the higher proportion of truncated CERP2 copies compared to CELE2 in *C. elegans* (fig. S4A). We also observed a high number of tandem m2+m1+ pairs in *Caenorhabditis becei* and *Caenorhabditis monodelphis* with different spacing between m1 and m2 sequences compared to CELE2, suggesting that other related TEs likely underwent expansion in these *Caenorhabditis* species (fig. S4B). These data indicate that the CERP2 and CELE2 expansions took place at different times in the *Caenorhabditis* clade, seeding thousands of m1m2 sequences and generating a large reservoir of potential regulatory elements.

HIM-17 predates the *Caenorhabditis*-specific expansions of CERP2 and CELE2, as orthologs could be identified not only in *Caenorhabditis* genomes but also in other *Eurhabditis* nematodes, with the exception of *Diploscapter* species (Fig. 3C and fig. S4, C and D). In light of the regulation of m1m2-associated promoters by HIM-17, we speculated that HIM-17 sequence might have undergone changes in line with the timing of the *Caenorhabditis* CERP2 expansion. Evolutionary analyses indicate that *him-17* underwent positive selection before divergence of the *Caenorhabditis* genus (branch-site test, *P* = 0.0007) in the same period in which the expansion of the CERP2 sequence also took place. Fourteen of the 34 sites under positive selection are located within its six THAP domains (Fig. 3D), which are related to the DNA binding domain of the *Drosophila* P-element transposase (*26*) and conserved in almost all HIM-17 orthologs. Moreover, across the *Caenorhabiditis* clade, the fourth THAP domain has higher similarity to the Pfam THAP consensus than the second THAP domain, whereas the opposite is true for the Strongylida clade (Fig. 3D). As these conserved changes in putative DNA binding domains occurred in parallel with the CERP2 expansion, we speculate that they may have enhanced HIM-17 recognition of the MITE-derived m1m2 motifs.

**Fig. 4.**
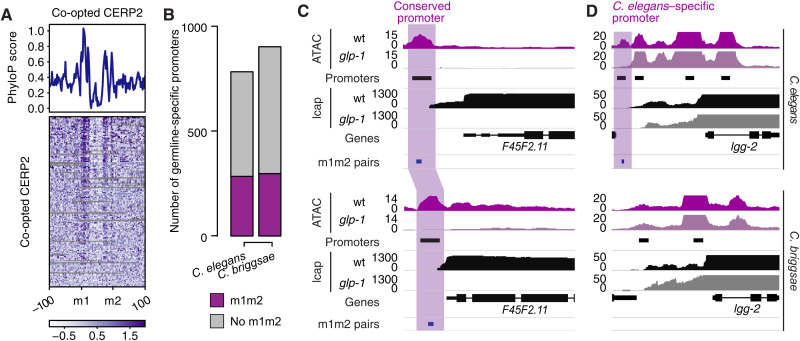
Evolutionary conservation and turnover of co-opted MITEs. (**A**) phyloP score profile (top) and heatmap (bottom) measured at germline-specific promoters associated to a divergent m1m2 pair in *C. elegans*. Elements not aligned to other species were removed from the heatmap. (**B**) Number of germline-specific promoters annotated in *C. elegans* and *C. briggsae*. (**C** and **D**) Examples of orthologs with a germline-specific CERP2-derived promoter in *C. elegans* conserved in *C. briggsae* (C) or *C. elegans* specific (D).

A large fraction of CERP2-derived promoters showed evidence of evolutionary conservation across multiple species, as indicated by peaks of phyloP scores (Fig. 4A). In particular, the m1 and m2 sequences showed elevated phyloP scores compared to neighboring sequences, indicative of purifying selection acting on these essential motifs. To directly evaluate and quantify whether co-option events in the *Caenorhabditis* genus have given rise to shared and/or lineage-specific regulatory elements, we analyzed germline promoters in *Caenorhabditis briggsae*, which diverged from *C. elegans* between ~20 and 100 million years ago (*30*, *31*). As for *C. elegans*, we identified *C. briggsae* germline–specific promoters by generating ATAC-seq and nuclear RNA-seq data from wild-type and a germline-less *C. briggsae glp-1* temperature-sensitive mutant that we generated using CRISPR editing (see Materials and Methods; fig. S5A).

As in *C. elegans*, we observed that *C. briggsae* germline–specific promoters are enriched for m1m2 pairs (Fig. 4B and fig. S5B). To evaluate the evolutionary conservation of the CERP2 co-opted promoters, we identified 1:1 orthologs associated with a co-opted promoter either in *C. elegans* (*n* = 327) or in *C. briggsae* (*n* = 322; table S1), including both germline-specific and nongermline-specific promoters. We found that 53% of the orthologs in each species were regulated by an evolutionary conserved co-opted promoter, and a further 22 to 27% had some evidence of conservation, indicated either by a promoter with only m1 or m2 or by an m1m2 pair not annotated as a promoter (see Materials and Methods). Thus, 53 to 80% of CERP2 co-option events are conserved in *C. elegans* and *C. briggsae* (Fig. 4C, fig. S5C, and table S1). The remaining 20 to 25% of the ortholog pairs had a co-opted promoter in only one species (Fig. 4D, fig. S5C, and table S1). This considerable evolutionary turnover could be explained either by the species-specific co-option of new or ancestral MITEs or by the degeneration of ancestral m1m2 sequences.

## DISCUSSION

Our work provides functional evidence for the large-scale co-option of TEs as germline-specific promoters in *Caenorhabditis*. Hundreds of co-opted promoters have been preserved by selection for many millions of years, demonstrating that TEs can have a profound impact on the host regulatory landscape. These ancient co-option events were detectable and traceable because MITEs containing the long m1-m2 motifs are still abundant in *Caenorhabditis* genomes. It is likely that co-option events involving parental TEs that have since degenerated or where the co-opted regulatory motifs are short would be missed because little evidence may remain. Thus, although it is impossible to determine the full scope of regulatory sequences that TEs have contributed to eukaryotic genomes, there is potential that a large fraction originates from TEs.

## MATERIALS AND METHODS

### Strains and growth conditions

*C. elegans* strains were cultured using standard methods (*32*). A complete list of strains is presented in table S1.

### Generation of a *C. briggsae glp-1(ts)* allele

CRISPR-Cas9 genome editing was used to generate the *C. briggsae glp-1(ts)* strain. Injections were performed in the wild-type (AF16) *C. briggsae* strain using guide RNA–Cas9 ribonucleoprotein complexes preassembled in vitro with in-house made Cas9 protein (*33*, *34*). Trans-activating CRISPR RNAs (tracrRNAs), CRISPR RNAs (crRNAs), and Ultramer repair templates were purchased from Integrated DNA Technologies. crRNAs were designed using the online CRISPOR tool (*35*). We engineered two different mutations in the *C. briggsae glp-1* gene, R955C (GCA → TCT) and G1036E (GGA → GAA), to mimic the *C. elegans* temperature-sensitive *e2141* and *q231* alleles, respectively. Single mutants did not display germline defects, but each produced some dead eggs at 27°C. We thus generated a double mutant, *glp-1(we58)*, carrying both R955C and G1036E. Double mutants were maintained at 16°C and failed to develop a germ line when grown from starved stage 1 larvae (L1s) at 27°C.

### Generation of *C. briggsae* ATAC-seq and nuclear RNA-seq data

Wild-type AF16 *C. briggsae* or *glp-1(we58)* mutants were grown in liquid culture from the starved L1 to the young adult stage using standard S-basal medium with HB101 bacteria (wild type at 20°C, *glp-1* at 27°C), frozen in liquid nitrogen, and stored at −80°C until use. Nuclei were isolated, and ATAC-seq and nuclear RNA-seq libraries were generated from wild-type and *glp-1(we58) C. briggsae* young adults as in (*20*). ATAC-seq and RNA-seq libraries were generated using approximately 1 million nuclei and for two biological replicates for each *C. briggsae* strain.

### HIM-17 ChIP-seq

HIM-17 ChIP-seq libraries were prepared from two biological replicates following the protocol described in (*36*). Briefly, frozen young-adult worms were ground to a powder, which was incubated in 1.5 mM ethylene glycol bis(succinimidyl succinate) (EGS) (Pierce 21565) in phosphate-buffered saline (PBS) for 8 min, followed by the addition of formaldehyde to a final concentration of 1%, and incubated for a further 8 min. The fixation was quenched for 5 min by the addition of 0.125 M glycine. Fixed tissue was washed 2× with PBS with protease inhibitors (Roche EDTA-free protease inhibitor cocktail tablets 05056489001) and once in FA buffer [50 mM Hepes (pH 7.5), 1 mM EDTA, 1% Triton X-100, 0.1% sodium deoxycholate, and 150 mM NaCl] with protease inhibitors (FA^+^) and then resuspended in 1 ml of FA^+^ buffer per 1 ml of ground worm powder. The extract was sonicated to an average size of ~250 bp using a Bioruptor Pico (Diagenode), and 20 μg of DNA was used per ChIP reaction. ChIP DNA was blunt ended, A-tailed, ligated to adaptors, amplified by PCR, and then size-selected using AMPure beads. Sequencing was performed on an Illlumina HiSeq 1500 machine using TruSeq adaptors.

### RNA sequencing

For each of two replicates, approximately 100 wild-type or *him-17(me24)* (m+z−; inherited wild-type maternal gene product and homozygous mutant for *him-17*) young adults grown at 20°C from the starved L1 stage were collected. *him-17(me24)* was derived from *him-17(me24)/tmC12 [tmIs1194]* mothers. Total RNA was extracted using TRIzol. Poly(A) RNA was isolated using the NEBNext Poly(A) mRNA Isolation Kit, and libraries were prepared using the NEBNext Ultra Directional RNA Library Prep Kit (E7760S).

### Processing of sequencing data

ChIP-seq data generated in this study, ATAC-seq data from isolated L1 PGCs (Gene Expression Omnibus (GEO) accession: GSE100651] (*37*), and ATAC-seq data from adult germ lines (GEO accession: GSE141213) (*20*) were preprocessed using trim galore (version 0.6.4; available at https://github.com/FelixKrueger/TrimGalore) and mapped using bwa mem (version 0.7.17) (*38*). Read depth-normalized coverage tracks from mapq10 reads were generated using MACS2 (*39*) (version 2.1.2; for ATAC-seq data processing, we used the following parameters: --nomodel –extsize 150 –shift -75), converted to bigWig format, and replicate pairs were used as input to identify peaks with the yapc software (version 0.1; available at https://github.com/jurgjn/yapc) (*40*), with –smoothing-window-width set to 100. Peaks passing an irreproducible discovery rate cutoff of 0.00001 (for ChIP-seq) or 0.001 (for ATAC-seq) were used in this study. ATAC-seq and HIM-17 ChIP-seq heatmaps were generated using the computeMatrix and plotHeatmap functions from the deepTools2 suite (version 3.4.3) (*41*). RNA-seq data were aligned on the genome using STAR (version 2.7.5a) (*42*) to generate coverage tracks. Gene expression was estimated using kallisto (version 0.46.2) (*43*).

### Genome annotation

Genome, gene, and protein annotations were downloaded from the repositories listed in table S1. For each protein coding gene, we extracted the genomic and protein sequences of its longest transcript. Repeats from Dfam (release 3.1) (*44*) were annotated in the *C. elegans* genome using the dfamscan.pl script available on the Dfam website. Repeat coordinates are available in table S1.

### Identification of germline-specific accessible sites in *C. elegans* and *C. briggsae*

Accessible sites in *C. elegans* and *C. briggsae* were identified using ATAC-seq data generated from wild-type and *glp-1* mutant strains. Single-end ATAC-seq reads were mapped on the respective genome assembly (WS275 for both *C. elegans* and *C. briggsae*) using bwa-backtrack (*45*), keeping only reads with high mapping quality (MAPQ) > 10] on fully assembled chromosomes. Coverage-normalized tracks generated using MACS2 were used as input for yapc to identify open chromatin regions. To annotate germline-specific accessible sites in each species, we compared ATAC-seq signals in wild-type and *glp-1* data using DiffBind (version 2.10.0) (*46*). We defined sites as germline-specific when the *glp-1* versus wild-type log_2_ fold change (LFC) < −2 and the adjusted *P* value (p.adj) < 0.01.

### Annotation of germline-specific promoters in *C. elegans* and *C. briggsae*

Germline-specific accessible sites were annotated as promoters in the *C. elegans* or the *C. briggsae* genomes, using a slightly modified version of the annotation pipeline from (*40*), based on patterns of nuclear RNA-seq data, which identifies regions of transcription elongation. Most *C. elegans* genes are trans-spliced (*47*), during which the original 5′ “outron” sequence is replaced by a short 22-nt leader sequence. Transcript start annotations in Wormbase predominantly mark the site of trans-splicing, not the site of promoters. As transcription of outron sequences is visible in nuclear RNA-seq data, promoters can be annotated on the basis of transcription from an accessible site to the annotated first exon of a gene. In this work, mapped RNA-seq reads from both replicates of each strain were randomly and evenly distributed in two pseudoreplicates to compensate for different sequencing depths. Accessible sites were annotated as promoters when (i) nuclear RNA-seq signal connected the site to an annotated first exon, allowing gaps in RNA-seq signal of up to 200 bp, and (ii) where a significantly higher RNA-seq signal was present in the regions +75 to +350 bp from the midpoint of an open chromatin region (relative to the downstream gene) compared to the −75- to −350-bp sequence.

### Motif enrichment and motif pair annotation

We used the MEME suite (version 5.0.5) (*48*) to identify motifs enriched in germline-specific promoters in *C. elegans* or *C. briggsae* (enrichment compared to non–GL-specific promoters, MEME-ChIP parameters used: -meme-nmotifs 6 -meme-minw 5 -meme-maxw 20). Enriched motifs were mapped on the genomes of different species with FIMO (*P* < 0.0005). We annotated all occurrences of m1 and m2 motifs separated by 10 to 30 bp—a range including the most frequently observed m1-m2 spacings—as m1m2 motif pairs and distinguished them based on the relative motif orientation into four arrangements: convergent m1m2, divergent m1m2, tandem m1+m2+, and tandem m2+m1+ (table S1).

### CERP2 and CELE2 enrichment at germline promoters

Significant enrichment of m1m2-containing CERP2 and CELE2 elements within germline-specific promoters was tested by permutation (*n* = 1000). Random repeat locations were defined using shuffleBed from BEDTools (*49*) by excluding gene bodies and maintaining the original chromosomal distribution.

### Analysis of the evolutionary relationships between m1m2 promoters and MITE elements

To determine the similarity between m1m2 pair–containing promoters and annotated CERP2 or CELE2 elements, we extracted the sequences of all divergent m1m2 (intermotif spacing: 12 to 16 bp) and tandem m2+m1+ pairs (intermotif spacing: 23 to 28 bp) plus 50-bp up-/downstream of each pair (orientation defined by m1) and then subjected the divergent m1m2 and tandem m2+m1+ sequences to separate multiple sequence alignments using MAFFT (with settings: --retree 1 –treeout –globalpair) (*50*), which produces a guide tree using a modified Unweighted Pair Group Method with Arithmetic Mean (UPGMA) method; similar results were obtained using other tree building methods. Motif pairs were annotated on the basis of overlap with promoters or annotated CERP2 or CELE2 MITE elements, giving four categories: promoter, MITE, promoter + MITE, and neither promoter nor MITE. Divergent m1m2 and tandem m2+m1+ output trees were plotted using the R package ape (*51*).

### Assessment of CERP2- and CELE2-derived promoter activity

Transgenes containing the annotated CERP2-associated promoter of *C16A11.4* or the CELE2-associated alternative promoter of *fat-1* upstream of *his-58::gfp::tbb-2* 3′ untranslated region (3′UTR) were generated using Mos-1–Mediated single-copy insertion (MosSCI) (*52*). Wild-type and mutant versions in which motifs were scrambled were generated. Promoter sequences used are given in table S1. Synthesized promoter sequences were ordered as plasmids containing att sites for Gateway cloning from GenScript, and reporter transgenes were constructed using three-site Gateway cloning (Invitrogen) using vector pCFJ150, which targets Mos site Mos1 (ttTi5605) on chromosome II (*52*), the promoter to be tested in site one, *his-58* in site two (plasmid pJA357), and *gfp-tbb-2* 3′UTR in site three (pJA256) (*53*). GFP signal was assessed using a Zeiss Axioplan microscope equipped with wide-field fluorescence microscopy. At least 20 individuals were scored per strain.

### *T05F1.2* promoter mutation

We used CRISPR-Cas9 to scramble the m1 and m2 sequences in the endogenous CERP2-associated promoter of *T05F1.2*. *T05F1.2* expression in the wild-type and mutant strains (*we59*) was quantified by qPCR using two different sets of primers and compared to *cdc-42* expression. Primer sequences used are available in table S1.

### Association of co-opted promoters with TF binding sites

ChIP-seq data from 283 *C. elegans* TFs were downloaded as aggregated peaks from the modERN website (https://epic.gs.washington.edu/modERN/) (*21*), and from these, we extracted only the data from 73 factors that were generated from young adult animals. We further included data from a single HIM-17 ChIP-seq replicate (3916_SDQ0801_HIM17_FEM2_AD_r1) available in modENCODE (*27*) but not included in Modern. The HIM-17 ChIP-seq reads were mapped on the ce11 genome using bwa-mem, and peaks were called using mapq10 reads with MACS2. For each factor, we compared the ratio of peaks overlapping germline-specific co-opted and non–co-opted promoters.

### Testing requirement for m1m2 motifs in HIM-17 chromatin association

To test whether HIM-17 requires motifs m1 or m2 for chromatin association at a co-opted promoter, three variants of the transgene driven by the CERP2-derived *C16A11.4* promoter were generated using MosSCI: scrambled m1, scrambled m2, or scrambled m1 and m2. ChIP-qPCR was performed for HIM-17, testing enrichment for the transgene promoter, for the co-opted *ztf-15* promoter as a positive control, and for two negative control loci showing no ChIP-seq enrichment for HIM-17. Experiments were done on three technical replicates from two biological replicates.

### *Him-17* gene expression analysis

DESeq2 (version 1.22.1) (*54*) was used to identify significantly up-regulated (LFC > 0, p.adj < 0.001) or down-regulated (LFC < 0, p.adj < 0.001) genes in *him-17* mutants compared to wild type. Gene Ontology enrichment analysis on differentially expressed genes was performed with clusterProfile (*55*). Direct targets were defined as genes significantly down-regulated in the mutant that have a HIM-17 ChIP-seq peak on their associated promoter. We evaluated transcription factor occupancy at promoters of direct target using curated modENCODE ChIP-seq peaks from 176 factors from Janes *et al.* (*40*).

### Annotation of CERP2 and CELE2 in different species

We extracted sequences from all CERP2 and CELE2 elements in *C. elegans* to refine Hidden Markov Models (HMMs) of these repeats using the HMMER3 suite (http://hmmer.org/). Fasta sequences of all repeats from each family were aligned against the CERP2 or CELE2 Dfam HMM using hmmalign (with parameter –trim). The resulting alignment was used to define new HMMs using hmmbuild. The HMMs were then used to annotate CERP2 and CELE2 repeats in nematodes with chromosome-level genome annotations (table S1) using nhmmer and requiring a minimal *E* value of 0.001.

### HIM-17 evolution and structure

HIM-17 orthologs were identified using BLASTP (*E* value < 0.00001) on the protein annotation from a number of nematode species (listed in table S1). To test the *him-17* sequence for positive selection, HIM-17 orthologs were aligned using MAFFT (*50*) with the L-INS-I method and then the output alignment was used to guide a codon-based alignment using PAL2NAL (*56*). The resulting alignment was used to test for positive selection acting on the common *Caenorhabditis* branch using the branch-site test (*57*) implemented in codeml from the PAML package (*58*). THAP domains in HIM-17 orthologs were annotated with hmmsearch using the THAP profile HMMs from the Pfam database (*59*).

### Analysis of co-opted promoters conservation

Sequence conservation of m1m2 pairs located in CERP2-derived germline-specific promoters was assessed using phyloP scores from 26 nematodes (phyloP26way from the cell release) available from the UCSC genome browser. To evaluate the conservation of individual CERP2 promoters in *C. elegans* and *C. briggsae*, we extracted all 1-to-1 orthologs (obtained from Wormbase) regulated by a co-opted promoter in at least one species, i.e., associated to at least one promoter containing an m1m2 pair in divergent orientation and spaced by 12 to 16 bp (CERP2-like arrangement). Co-opted promoters were defined as conserved when both orthologs were associated with a co-opted promoter. We considered co-opted promoters as potentially conserved when the ortholog in the other species was either (i) associated with a promoter containing at least m1 or m2 or (ii) when an m1m2 pair was located in the putative promoter region (−1000 bp/+200 bp) of the orthologs’ transcription start site but was not in an annotated promoter. When none of the criteria were met, we defined the co-opted promoter as species specific.
