# Supplementary material for: Widespread transposon co-option in the Caenorhabditis germline regulatory network
Source: Sci Adv. 2022 Dec 16;8(50):eabo4082. doi: 10.1126/sciadv.abo4082 (PMC9757741; doi:10.1126/sciadv.abo4082)
Supplement: Supplementary file 1 — Figs. S1 to S5 [file sciadv.abo4082_sm.pdf]

Science Advances
AAAS

Supplementary Materials for

Widespread transposon co-option in the *Caenorhabditis* germline regulatory network

Francesco Nicola Carelli *et al.*

Corresponding author: Francesco Nicola Carelli, fnc21@cam.ac.uk; Julie Ahringer, ja219@cam.ac.uk

Sci. Adv. **8**, eabo4082 (2022)
DOI: 10.1126/sciadv.abo4082

This PDF file includes:

Figs. S1 to S5
Table S1

Figure S1

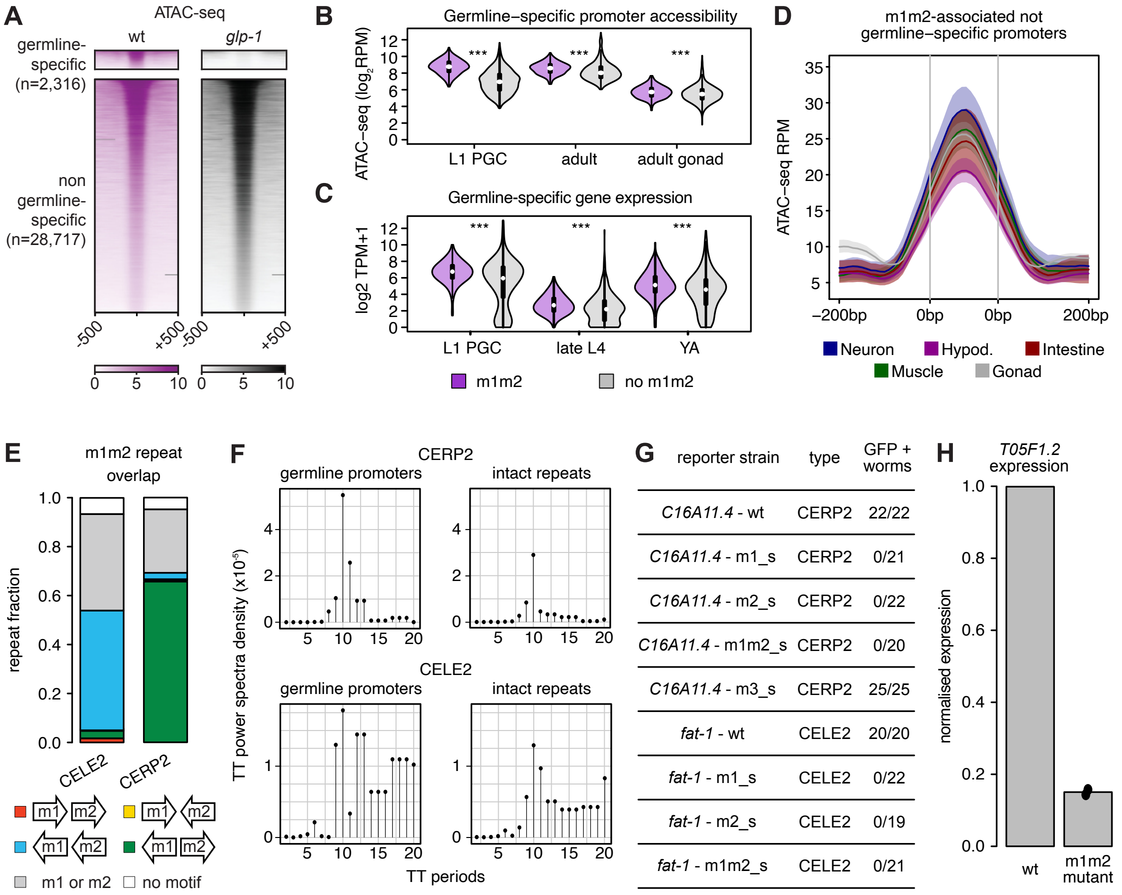

Fig. S1. TE enrichment at germline-specific elements in C. elegans. (**A**) ATAC-seq signal at *C. elegans* open chromatin regions. (**B**) ATAC-seq coverage at germline-specific promoters. (**C**) Expression levels of genes regulated by a unique germline-specific promoter (i.e. not associated with other alternative promoters). (**D**) ATAC-seq coverage from individual tissues (*18*) over non-germline-specific promoters associated with an m1m2 pair. Continuous lines: average; shaded areas: standard deviation. (**E**) Genome-wide fraction of CERP2 and CELE2 elements overlapping m1m2 pairs in any arrangement. (**F**) Power Spectral Densities of TT dinucleotides measured downstream of intact and germline-specific promoter-associated divergent m1m2 (top) and tandem m2+m1+ pairs (bottom). (**G**) Fraction of young adult hermaphrodites carrying indicated transgenes with GFP expression in the germ line. (**H**) Fold-change (measured by qPCR) in expression of the *T05F1.2* gene after mutating its promoter compared to wt.

Figure S2

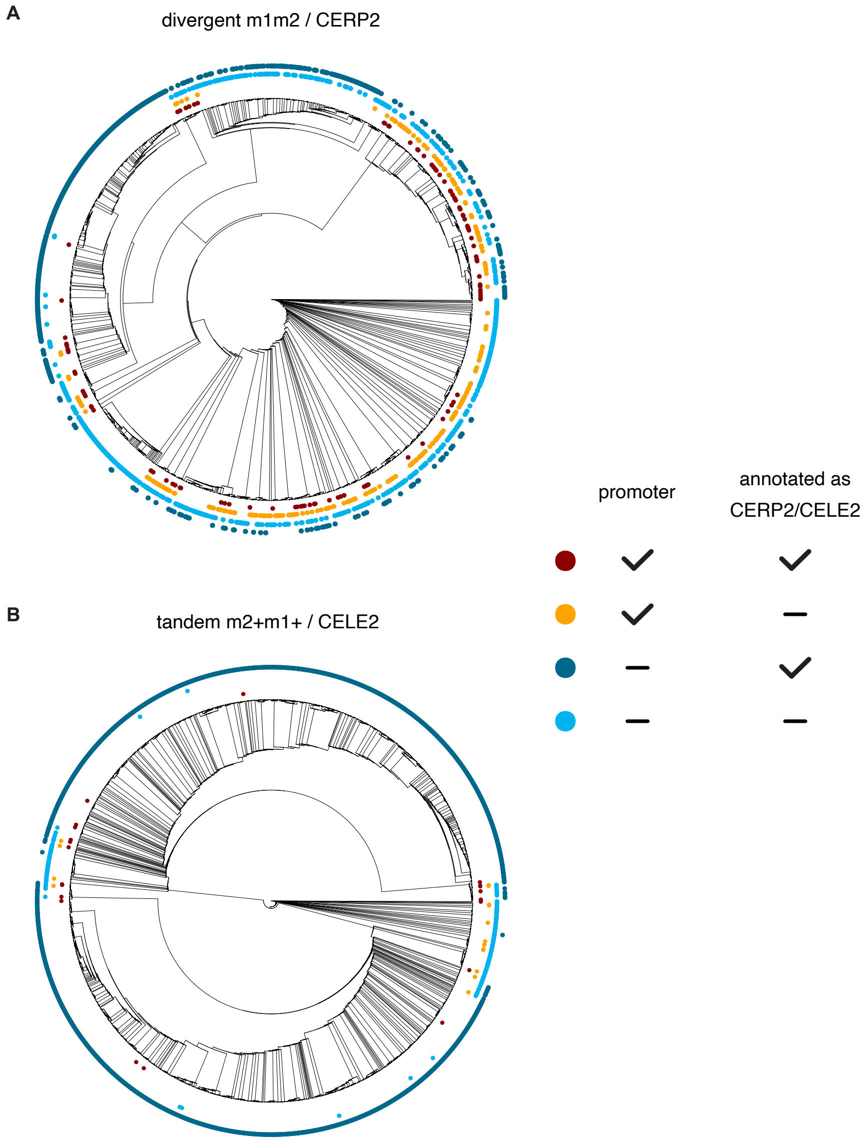

Fig. S2. Sequence similarity of divergent m1m2 and tandem m2+m1+ loci. Guide trees reconstructed based on the multiple alignment of divergent m1m2 (**A**) and tandem m2+m1+ (**B**) sequences (+ 50bp up-/downstream). Colour coded circles at the tip of each branch distinguish each m1m2 pair based on its promoter activity and annotation as a CERP2 (**A**) or CELE2 (**B**) repeat. Branch lengths not proportional to sequence divergence to allow the visualisation of sequence relationships.

Figure S3

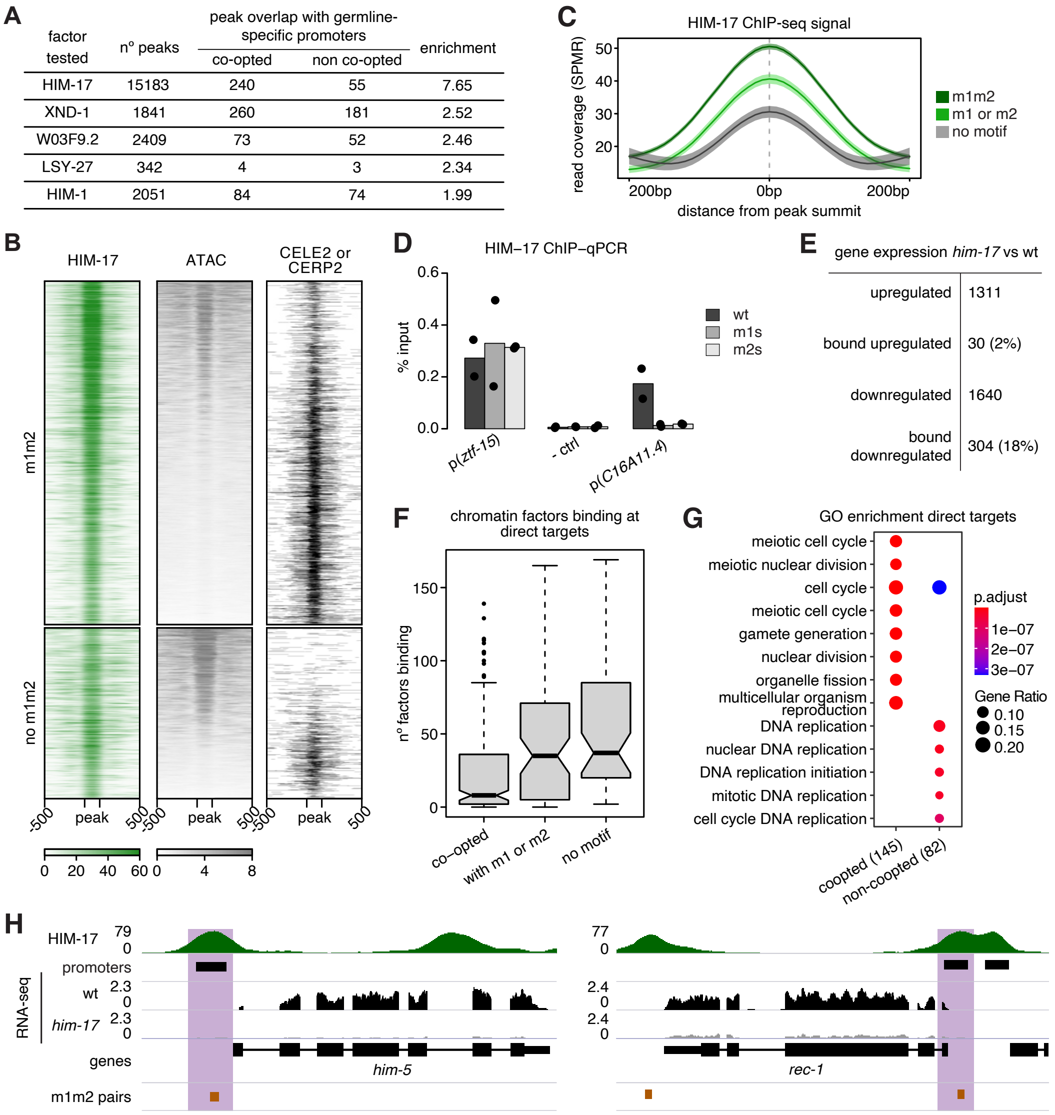

Fig S3 HIM-17 binds co-opted and inactive MITEs. (**A**) Summary statistics of top overlaps between co-opted and non-co-opted germline-specific promoters and the modERN/modENCODE peaks set. (**B**) HIM-17, ATAC-seq and CERP2 or CELE2 enrichment over HIM-17 peaks. Top, peaks overlapping an annotated m1m2 pair (n=2364); bottom, peaks without an annotated m1m2 pair (n=1175). (**C**) HIM-17 ChIP-seq signal at HIM-17 peaks overlapping m1m2 pairs, individual motifs or no motif. SPMR: signal per million reads (averaged across two replicates) (**D**) ChIP-qPCR enrichment of HIM-17 as % of input in strains containing the wt, m1 and m2 scrambled versions of the *C16A11.4* promoter integrated in the chrII MosSCI site (see Methods). Signal at endogenous co-opted promoter p(*ztf-15*) is shown as a positive control. (**E**) Number of upregulated and downregulated genes in *him-17* mutants. When the factor (in wt) overlapped any of the genes

differentially expressed in the mutant, the gene was considered a direct target. Percentages: fraction of direct targets over all DE genes. (**F**) Chromatin factor occupancy at promoters of direct targets. Promoters were distinguished into co-opted (i.e. those overlapping an m1m2 pair), those overlapping a single motif, and those overlapping no motif. (**G**) GO term enrichment of direct targets. (**H**) HIM-17 binding profile and RNA-seq profiles in wt and *him-17* mutants at the *him-5* and *rec-1* loci.

Figure S4

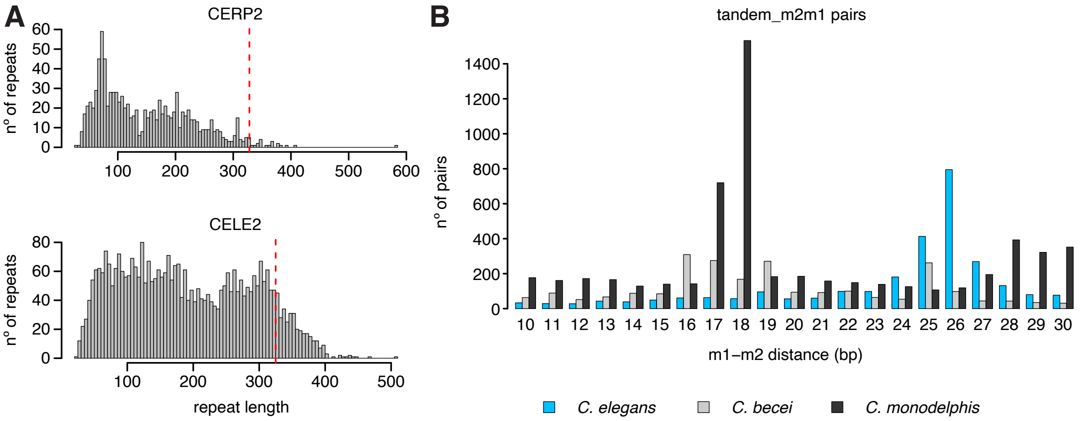

C

	Ortholog	id%	aln%
C. inopinata	Sp34_50272300	74.8	96.6
C. briggsae	WBGene00031009	71.2	97.6
C. nigoni	Cni-him-17	70.8	97.6
C. sinica	CSINI.Csp5_scaffold_01371.g20435	70.2	99.7
C. remanei	GCK72_017715	71.0	100
C. becei	CSP29.g2205	66.8	100
C. panamensis	CSP28.g43.t1	68.3	94.5
C. afra	CAFRA.g590	64.8	97.9
C. uteleia	CSP31.g17950	58.8	93.1
C. quiockensis	CSP38.g16121	60.1	89.4
C. virilis	CVIRI.g11655.t1	56.4	86.2
C. bovis	CBOVI.g1856	58.6	87.3
C. plicata	CPLIC.g13429	54.3	99.6
C. monodelphis	CMONO.g20231	44.9	99.6
H. placei	HPLM_0000813001	33.4	95.4
H. contortus	HCON_00162800	33.6	95.4
H. polygyrus	HPOL_0000104401	32.7	96.4
N. brasiliensis	NBR_0001102701	34.3	86.5
D. viviparus	DICVIV_08733	32.1	61.7
A. caninum	ANCCAN_00941	33.4	86.9
A. ceylanicum	Acey_s0007.g3522	32.5	98.0
O. tipulae	g06918	37.5	81.9
A. rhodensis	ARH_g3726	38.6	84.1
H bacteriophora	Hba_11030	37.3	64.6
P. pacificus	-	-	-
P. expectatus	-	-	-
P. redivivus	-	-	-

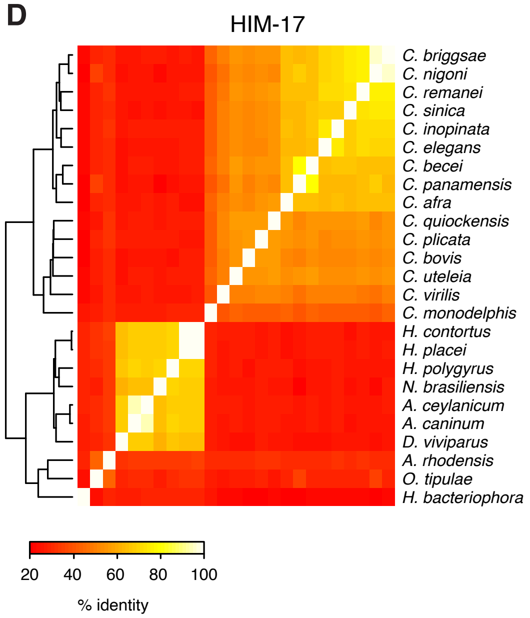

Fig. S4. Evolution of m1m2 pairs and their binding factor in nematodes. (**A**) Length of annotated CERP2 and CELE2 elements in *C. elegans*; red dashed line indicates length of consensus repeat sequence. (**B**) Spacing between m1 and m2 motifs in tandem_m2m1 in a subset of species. (**C**) HIM-17 orthologs in different nematodes, with % identity and % of alignment length with the corresponding *C. elegans* protein. (**D**) Pairwise % identity across HIM-17 orthologs.

Figure S5

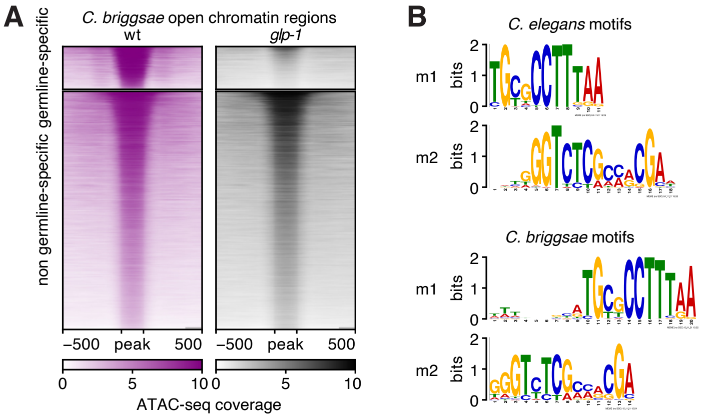

C

	Co-opted promoters	Conserved promoter	Conserved promoter (m1 or m2)	Not conserved
C. elegans	327	173	87	67
C. briggsae	322	173	71	78

Fig. S5. Evolutionary conservation and turnover of co-opted MITEs. (**A**) ATAC-seq signal (in RPM) from wild-type and *Cbr-glp-1* mutant over *C. briggsae* open chromatin regions. (**B**) Comparison of m1 and m2 motif logos in *C. elegans* and *C. briggsae*. (**C**) Summary of CERP2 promoter conservation between *C. elegans* and *C. briggsae* orthologs.

Table S1.

Table containing the following information:

1) Coordinates of accessible sites in *C. elegans*
2) Motif enrichment in *C. elegans* germline-specific promoters
3) Coordinates of repetitive elements from Dfam in the *C. elegans* genome
4) Coordinates of m1m2 pairs in *C. elegans*
5) Coordinates of HIM-17 ChIP-seq peaks in *C. elegans*
6) Motif enrichment in C. elegans HIM-17 peaks
7) Differential gene expression analysis in wild-type vs him-17 (DESeq2 output)
8) List of *C. elegans* HIM-17 direct targets
9) Coordinates of accessible sites in *C. briggsae*
10) Motif enrichment in *C. briggsae* germline-specific promoters
11) Coordinates of m1m2 pairs in *C. briggsae*
12) Conservation of *C. elegans* co-opted CERP2 promoters in *C. briggsae*
13) Conservation of *C. briggsae* co-opted CERP2 promoters in *C. elegans*
14) List of species investigated in this study and associated data source
15) Sequences of primers used in this study
16) List of strains used in this study
17) List of MosSCI constructs used in this study
